# Environmental and Yield Comparison of Quick Extraction Methods for Caffeine and Chlorogenic Acid from Spent Coffee Grounds

**DOI:** 10.3390/foods12040779

**Published:** 2023-02-10

**Authors:** Ibtissam Bouhzam, Rosa Cantero, Mercè Balcells, María Margallo, Rubén Aldaco, Alba Bala, Pere Fullana-i-Palmer, Rita Puig

**Affiliations:** 1 Department of Computer Science and Industrial Engineering, University of Lleida (UdL), Pla de la Massa, 8, 08700 Igualada, Spain; 2 Department of Chemistry, University of Lleida (UdL), Rovira _Roure 191, 25198 Lleida, Spain; 3 Department of Chemical and Biomolecular Engineering, University of Cantabria, Av. de Los Castros s/n, 39005 Santander, Spain; 4 UNESCO Chair in Life Cycle and Climate Change ESCI-UPF, Pg. Pujades 1, 08003 Barcelona, Spain

**Keywords:** life cycle assessment, supra solvents method, water extraction, spent coffee grounds circularity

## Abstract

This study aims to provide an overview of different extraction methods to obtain chlorogenic acid (CA) and caffeine (Caf) from spent coffee grounds (SCG). This overview shows that the quantity extracted is highly dependent on the type of SCG, so experiments using the same SCG are needed to compare different methods. Three easy and simple extraction methods will be tested at a laboratory scale and environmentally compared. All three experiments were of 1 min duration: first, using supramolecular solvent; second, with water and vortex; and third, with water assisted by ultrasound. Water extraction assisted by ultrasound at room temperature yielded the greatest quantity of chlorogenic acid and caffeine, with 1.15 mg CA/g and 0.972 mg Caf/g, respectively. Extraction using supra-solvent leads to a lower content of CA in the supra-phase since it has more affinity for the water-based inferior phase. An environmental assessment using life cycle assessment has been carried out to compare water and supra extraction methods for the manufacture of two different commercial products: a face cream and an eye contour serum. Results show that the type of solvent and the amount of active substance extracted have a great influence on the environmental results. The results presented here are important for companies willing to obtain these active substances at an industrial scale.

## 1. Introduction

Food waste is one of the important issues facing the world [1]. The impacts and the real value of food waste have not been fully understood. Most researchers have agreed that food waste is linked to economic, environmental, and social problems [2]. In 2020, 20% of global food production was wasted [3], and it is estimated that 8% to 10% of global greenhouse gas emissions are attributed to food waste [4]. Therefore, there is an urgent need for sustainable methods to reduce food waste [5,6,7].

Spent coffee grounds (SCG) are among the most common organic waste generated all around the world. Coffee is the most widely consumed product in the world after water; it is also ranked the second-traded commodity worldwide after petroleum [8]. According to the International Coffee Organization, the world coffee consumption for the year 2020/2021 reached around 166 million bags of 60 kg, of which Europe accounts for almost a third, generating a great number of residues every year [9]. The process of making coffee involves the formation of various by-products, including the coffee husk, pulp, silverskin, and spent coffee grounds (SCG). Spent coffee grounds (SCG), for example, are produced in large amounts during the process of instant coffee preparation (at home, in restaurants, bars, and other businesses in the food industry) and mostly discharged into the environment [10] or burned [8,11]. It has been calculated that one ton of green coffee beans converted into coffee beverages produces 650 kg of discarded coffee grounds [11].

Nonetheless, there has been a surging interest in recycling these residues in recent years, particularly in the last years when the number of papers published on the theme “waste coffee grounds” has increased because they include various components that could be useful in the food, cosmetics, and pharmaceutical industries, such as caffeine, carbohydrates, lipids, and phenolic acids such as chlorogenic acid (CA) [12,13,14].

Chlorogenic acid and caffeine have received considerable attention due to their interesting properties. CA’s health benefits for humans are being supported by an increasing body of scientific evidence. CA appears to work as a protective agent, in particular, preventing or lowering oxidative stress of cell structures and functions while also boosting health. CA has also been studied for its potential to improve blood pressure and glucose regulation [13,15,16]. Caffeine, which is the most important alkaloid of the coffee species, presents various biological activities, such as stimulation of the central nervous system, myocardial stimulation, and peripheral vasoconstriction. Some studies suggest that caffeine is effective in reducing weight through thermogenesis and fat oxidation, effective in reducing the effects of ultraviolet radiation and damage induced by free radicals on the skin, and also as an adjunct to the treatment of hair loss [17].

Chlorogenic acid, an important part of the phenolic compounds, and caffeine are present in spent coffee grounds [14]. Thus this residue has been suggested as a sustainable alternative to obtain these substances.

Some methods have been tested at a laboratory scale for their recovery using different solvents and techniques, such as liquid–solid extraction [18,19,20], Soxhlet extraction [21,22], autohydrolysis [23], ultrasound [21,24,25,26], microwave-assisted extraction [27,28], subcritical water extraction [29], pressurized liquid [12], hydrothermal treatment [8], and supramolecular solvent extraction [30].

Supramolecular solvents (SUPRAS) have been reported as an efficient alternative method to organic solvents, effective in solubilizing a variety of solutes, and a readily available method, making it a very attractive process for extraction applications. SUPRAS are nanostructured liquids generated from aqueous or hydro-organic colloidal dispersions of amphiphilic substances through spontaneous processes of self-assembly and coacervation [31].

Our final aim is to contribute to the promotion of circularity for the processing of spent coffee grounds (SCG) by providing comparable information on different extraction methods. The purpose of this study is to compare, for the first time, supramolecular solvent extraction with conventional methods to extract chlorogenic acid and caffeine. The comparison will include both extraction yields and environmental performance. In addition, an overview of the different reported extraction methods will be provided.

## 2. Literature Review

Different methods to obtain chlorogenic acids and caffeine from spent coffee grounds can be found in the literature (Table 1 and Table 2).

Chlorogenic acid is one of the phenolic compounds most commonly quantified in the literature studies. According to Andrade et al. [15], chlorogenic acid was the phenolic component found in higher concentrations in the extracts of coffee grounds. Gallic acid, p-hydroxybenzoic acid, protocatechuic acid, vanillic acid, and tannic acid were also found, but in lower amounts. Chlorogenic acids include three main groups: caffeoylquinic acids (CQAs: 3-CQA, 4-CQA, and 5-CQA), dicaffeoylquinic acids (diCQAs: 3,4-diCQA, 3,5-diCQA, and 4,5-diCQA), and feruloylquinic acids (FQAs: 3- FQA, 4- FQA, and 5-FQA) [32].

In only a few studies, researchers have evaluated the content of chlorogenic acids as 5-O-Caffeoylquinic acid (abbreviated as CA) or total chlorogenic acids as tCA (those including the different isomers). Most of them have extensively reported the total phenolic compounds as gallic acid equivalents (GAE). The total phenolic content can be measured using the Folin–Ciocalteu method, and results are expressed as gallic acid equivalents (GAE).

The Folin–Ciocalteu method, developed by Singleton et al. [33], was first employed to determine “wine tannin” level, then gained widespread acceptance and is being used on a variety of plant materials and foods [34]. The principle of the method relies on the reduction of the Folin–Ciocalteu reagent in the presence of an antioxidant. Thus, the reductive capacity increases with the concentration of the phenolic compounds [35]. Calibration curves using various chemical standards should be used when calculating the total phenolic content. The most commonly used standard is gallic acid, which has been widely used in a variety of fruits, vegetables, red wines, and plant extracts, including berries, herbs, cereals, tree materials, plant sprouts, seeds, and spent coffee grounds [18,19,23,34].

Table 1 presents studies conducted by different authors, which focus on extracting caffeine and chlorogenic acid through different methods. However, the results are difficult to compare because the coffee sample, the solvent/solid ratio, and the way to measure the results are different. Nevertheless, some conclusions from the literature review will be noted. Results are usually quantified in three different ways: gallic acid equivalents (GAE), which measure the total phenolic compounds; chlorogenic acid (CA); and caffeine, which specifically quantifies those analytes.

The most common solvent used in the literature was ethanol in different proportions, with high variation in the solvent/SCG ratio (from 5 to 83 mL/g). The recovery rate of the total phenolic compounds ranged from 17 to 273 mg GAE/g, with an extraction time varying from 5 min to 6 h (Table 1), except for the supramolecular method, which only takes 1 min. The highest content was obtained by the Soxhlet method using ethanol (273.34 mg GAE/g). Water also successfully accomplished the extraction of high amounts of phenolic compounds; around 86.23 mg GAE/g was extracted with subcritical water extraction (SWE), also known as pressurized hot water extraction. SWE has emerged as a viable method for extracting bioactive and nutritional components because it has several advantages, including reduced organic solvent use, excellent extraction efficiency, and an environmentally friendly technology [29]. Autohydrolysis, pressurized liquid, and hydrothermal pretreatment have also been demonstrated to be effective methods for recovering phenolic compounds from spent coffee grounds [12,23].

Chlorogenic acid can be measured as CA (using 5-O-Caffeoylquinic acid as standard) or as total chlorogenic acid (tCA) when all the peaks corresponding to the different isomers of chlorogenic acid are integrated. Results found in the literature (expressed as mg of analyte per gram of dry SCG) have a wide range of variation, from 0.02 mg CA/g to 5.97 mg tCA/g (Table 1 and Table 2).

Caffeine (1, 3, 7-trimethylxanthine) is also the most desired compound to isolate from spent coffee grounds. The maximum contents reported in the literature ranged from 3.32 to 11.50 mg Caf/g in mixtures of Arabica and Robusta varieties (Table 1 and Table 2). The recovery rate may vary in function of the type of coffee variety, as said before. According to Campos-Vega et al. [36], the caffeine content in Robusta is almost twice (1.4–2.9%) compared with Arabica (0.9–1.6%) or a Robusta mix (60 Arabica/40 Robusta) (1.7%).

Several studies [12,18,23,29,37] (see Table 1) have tried to optimize condition parameters (time, temperature, and solvent ratio) for the same extraction method and solvent using statistical tools, such as response surface methodology or chemometrics in general.

**Table 1:** Extraction methods and conditions reported in the literature.

Extraction Type	Solvent	Time	Temperature	Ratio Solvent/Solid	Results	Ref.
Hydrothermal pretreatment	Water	20 min	120 °C	20 mL/g	32.92 mg GAE/g	[8]
Pressurized liquid	Ethanol 25–75%	5–20 min	195 °C	-	22.91 mg GAE/g 9.66 mg Caf/g	
						[12]
Solid/liquid	Ethanol 70%	2 h	50 °C	40 mL/g	17.09 mg GAE/g (Espresso)	[18]
Solid/liquid	Ethanol 70%	2 h	50 °C	40 mL/g	19.98 mg GAE/g (Capsules)	[18]
Solid/liquid	Ethanol 60%	30 min	60 °C	50 mL/g	28.26 mg GAE/g	[19]
Soxhlet	Ethanol 50%	-	Ambient	5 mL/g	273.34 mg GAE/g	[22]
Autohydrolysis	Water	50 min	200 °C	15 mL/g	40.36 mg GAE/g	[23]
Solid/liquid	Ethanol 60%	8 h	25 °C	20 mL/g	0.54 mg CA/g	[24]
Soxhlet	Ethanol 60%	6 h	-	25 mL/g	0.63 mg CA/g	[24]
Ultrasound	Ethanol 60%	1 h	50 °C	20 mL/g	0.93 mg CA/g	[24]
Ultrasound	Ethanol	34 min	40 °C	17 mL/g	33.84 mg GAE/g 1.43 mg CA/g	[25]
Subcritical water	Water	55 min	177 °C	71 mL/g	86.23 mg GAE/g	[29]
Subcritical water	Water	30 min	170 °C	50 mL/g	1.41 mg CA/g	[29]
Supramolecular solvent	1-hexanol 24%	1 min	Ambient	5.7 mL/g	4.3 mg CA/g 3.32 mg Caf/g	
	Ethanol 30%					[30]
	Water 46%					
Solid/liquid	Ethanol 25%	15 min	60 °C	83 mL/g	0.02 mg CA/g (Marcilla coffee)	[37]
Solid/liquid	Ethanol 25%	15 min	60 °C	83 mL/g	0.8 mg CA/g (Arabica coffee)	[37]

CA: Chlorogenic acid (measured from one single chromatographic peak, the one of the standard 5-O-Caffeoylquinic acid); GAE: gallic acid equivalent (measuring the total phenolic compounds); and Caf: caffeine (measured using the standard 1,3,7-trimethylxantine). The color is used to easily identify the results/methods described in the same reference and distinguish from the others. The extraction types belong to the same reference (and probably same SCG) when marked with the same color.

Only a few studies, those represented in Table 2, have compared extraction methods and solvents using the same spent coffee ground.

Andrade et al. [15] have tested CO_2_ supercritical fluid extraction (SFE CO_2_), which is known as a decaffeination process to extract caffeine. The results are expressed in terms of concentration (mg per g of extract). Using this method, Andrade et al. [15] achieved an extraction rate of 41.3 mg/g of extract at 333.15 K and 300 bar. Regarding low-pressure methods, ultrasound gave a higher concentration (38.2 mg/g extracts) than Soxhlet (25.9 mg/g extracts) (Table 2). The solvent dichloromethane recovered the most caffeine (38.2 mg/g extracts), followed by ethanol (25.7 mg/g extracts), and then hexane (0.734 mg/g extracts) with ultrasound extraction [15] (Table 2). According to Shalmashi et al. [38], caffeine’s solubility decreases in the following order: chloroform, dichloromethane, acetone, ethyl acetate, water, methanol, ethanol, and carbon tetrachloride. Hence, the polarity of the solvent has a significant impact on the extraction level of caffeine.

Vandeponseele et al. [11] extracted higher content of caffeine (4.32 mg Caf/g) using an efficient combination of ethanol (40%) with water than pure water (3.63 mg Caf/g).

Regarding chlorogenic acid, some investigations have compared ultrasound with high hydrostatic pressure on the extraction of phenolics (measured as chlorogenic acid, CA). The greatest recovery rate of chlorogenic acid was achieved using ultrasound, which gave a slightly greater yield than high hydrostatic pressure [39] (Table 2).

Concerning the influence of the solvent, Panusa et al. [19] have detected small differences between the use of pure water (5.67 mg tCA/g) and ethanol 60% (5.97 mg tCA/g) in SCGs collected from bars, as well as in coffee capsules (2.15 mg tCA/g for pure water) and (2.26 mg tCA/g for ethanol 60%), which also revealed that SCGs from bars are twice as rich in chlorogenic acid as capsules residue (see Table 2).

**Table 2:** Comparison of the extraction methods and solvents for the extraction of caffeine and chlorogenic acid using the same SCG.

Extraction Methods	Solvent	Time	Temperature	Ratio Solvent/ Solid	Results	Ref.
Solid/liquid	Ethanol 40%	15 min	20 °C	25 mL/g	4.32 mg Caf/g	[11]
	Water 100%				3.63 mg Caf/g	
	Ethanol 100%				0.26 mg Caf/g	
Ultrasound extraction	Hexane				0.734 mg Caf/g extract	
	Dichloromethane	2 h	Ambient T	30 mL/g	38.2 mg Caf/g extract	
	Ethanol				25.7 mg Caf/g extract	
Soxhlet	Hexane				3.27 mg Caf/g extract	[15]
	Dichloromethane	6 h	Ambient T	30 mL/g	25.9 mg Caf/g extract	
	Ethanol				11.8 mg Caf/g extract	
SFE CO_2_	CO_2_	4.30 h	333.15 K (200 bar)	-	27.2 mg Caf/g extract	
	CO_2_	4.30 h	333.15 K (300 bar)	-	41.3 mg Caf/g extract	
SFE CO_2_ + ethanol	CO_2_ + ethanol	4.30 h	333.15 K	-	23.4 mg Caf/g extract	
Solid/liquid	Water 100%	30 min	60 °C	50 mL/g	5.67 mg tCA/g 11.23 mg Caf/g (Arabica 80%, Robusta 20%)	[19]
	Ethanol 60%				5.97 mg tCA/g 11.50 mg Caf/g (Arabica 80%, Robusta 20%)	
Ultrasound	Methanol 80%	15 min	25 °C	10 mL/g	0.085 mg CA/g	[39]
High hydrostatic pressure					0.081 mg CA/g	

CA: Chlorogenic acid (measured from one single chromatographic peak, the one of the standard 5-O-Caffeoylquinic acid); tCA: total chlorogenic acid (measured integrating all the peaks corresponding to the different chlorogenic acid isomers); and Caf: caffeine (measured using the standard 1,3,7-trimethylxantine).). The color is used to easily identify the results/methods described in the same reference and distinguish from the others. The extraction types belong to the same reference (and probably same SCG) when marked with the same color.

Thus, the recovery rate of CA and caffeine extracted depends a great deal on the composition of SCGs, which is affected by the type of coffee bean, the roasting condition, and the coffee-making process [40,41,42,43,44].

Many studies have reported that the caffeine/CA ratio directly increases with the roasting intensity of the coffee bean [43]. In addition, it depends on the extraction method and conditions used (solvent type, solid/solvent ratio, temperature, pressure, and extraction time) [30]. This is why it is important to determine the most convenient solvent and extraction method for caffeine and CA extraction using the same sample of SCG.

## 3. Materials and Methods

In this section, the materials and methods for the three experimental extraction methods tested will be presented.

### 3.1. Chemicals and Reagents

1-hexanol (98%), ethanol (99.9%), and glacial acetic acid (99.8%) were purchased from Scharlab (Barcelona, Spain). Methanol (99.9%) was supplied by ITW reagents (Barcelona, Spain). Caffeine (1,3,7-trimethylxantine, HPLC grade) and 5-chlorogenic acid (5-O-Caffeoylquinic acid, 5-CA, 98%) were supplied by Sigma-Aldrich (St. Louis, MO, USA).

All chemicals were analytical reagent grade. Ultrapure water was prepared using a Milli-Q System equipped with a 0.22 µm filter purchased from Merck Millipore (Burlington, MA, USA).

### 3.2. Equipment

Quantification of caffeine and 5-CA was carried out using a high-performance liquid chromatograph (HPLC) coupled to a Waters 2996 UV Detector (Milford, MA, USA). The stationary phase was an Ultra C8 column (5 µm particle size, 150 mm length, 4.6 mm i.d.) from Scharlab (Barcelona, Spain). All data were processed using the Empower Solutions Software (Orlando, FL, USA).

A Velp Scientifica F202A0176 vortex shaker (Monza et de la Brianza, Italy), a J.P. Selecta 7002575 centrifuge, and a 3000865 ultrasound bath (Barcelona, Spain) were used for sample preparation.

### 3.3. Spent Coffee Grounds

Spent coffee grounds were provided by two restaurants (SCG1 and SCG2) located in the municipality of Igualada in the province of Barcelona, Spain. Both samples were derived from mixtures of Arabica and Robusta coffee varieties. The roasted coffee beans were from different producers (SCG1, “Novell gourmet Responsable”, and SCG2, “Cafes MamaSame”, with SCG1 being less roasted than SCG2). Both SCGs were obtained after the preparation of coffee using an espresso machine. The mixtures were manually homogenized, naturally dried, and stored in capsules at room temperature for further extractions. Experiments were performed in triplicate.

### 3.4. Extraction with Supra Solvents

The so-called supra solvents are a mixture of water with two additional solvents. In the present case, 1-hexanol, ethanol, and water were used. In the first step, a colloidal suspension of ethanol and 1-hexanol was formed. In the second step (coacervation), water was added, giving rise to a separation phenomenon of liquid phases: the superior (supra) and the inferior phases [31].

The extraction experiments were carried out according to the principle of the SUPRAS method mentioned above. A sample of 0.7 g of dry SCG was mixed with ethanol (1.2 mL), 1-hexanol (0.96 mL), and water until a total volume of 4 mL was obtained. The whole solution was vortex-shaken for 1 min at 3000 rpm and then centrifuged for 30 min at 4200 rpm. The volume of SUPRAS produced was calculated from the cylindrical volume formula πr^2^h, where r is the radius of the tube and h is the height of the supra phase of the extract. The height was measured using a digital caliper. The resulting supra solution was filtrated with a syringe filter (0.45 μm) before HPLC analysis. Experiments were performed in triplicate.

### 3.5. Extraction with Water

The extraction method with water was performed by adding 0.7 g of SCG to 4 mL of ultrapure water. The tube with the mixture was vortex-shaken at 3000 rpm or immersed in an ultrasound bath (50/60 Hz, 195 W) for 1 min, then centrifuged for 30 min at 4200 rpm and filtrated with a syringe filter (0.45 μm) before HPLC analysis. Experiments were performed in triplicate.

### 3.6. Analysis of Caffeine and Chlorogenic Acid by HPLC-UV

The mobile phase was prepared with ultrapure water and methanol, which were both acidified with 0.1% (*v*/*v*) of HPLC-grade acetic acid, and under a constant flow of 0.6 mL/min. The elution gradient started with 18% methanol and 82% ultrapure water and ended with 30% methanol and 70% ultrapure water for 15 min. The sample injection volume was 20 µL at 36 °C, and the detection was performed with a UV/Visible diode at a wavelength of 278 nm (for both chlorogenic acid and caffeine detection). Quantitative analysis was conducted by external calibration using standard solutions of 5-O-Caffeoylquinic acid and caffeine prepared in ultrapure water in concentration ranges from 1 mg/L to 200 mg/L and 100 mg/L to 600 mg/L, respectively.

## 4. Experimental Results and Discussion

All the results are expressed as the arithmetic mean of three replicates and their coefficients of variation. The coefficient of variation is the standard deviation divided by the arithmetic mean. The standard deviation measures the dispersion of the three values obtained with respect to the mean (higher dispersion of values and higher standard deviation).

### 4.1. Extraction with Supra Solvents Using Vortex

The capacity of SUPRAS to extract caffeine (Caf) and chlorogenic acid (CA) was previously reported in the literature, using 1-hexanol as an amphiphile and ethanol:water as a hydro-organic media [30]. The ratio of 1-hexanol/ethanol (24/30, *v*/*v*) has been reported to give the best results. Therefore, all the experiments were performed using this ratio.

**Figure 1:**
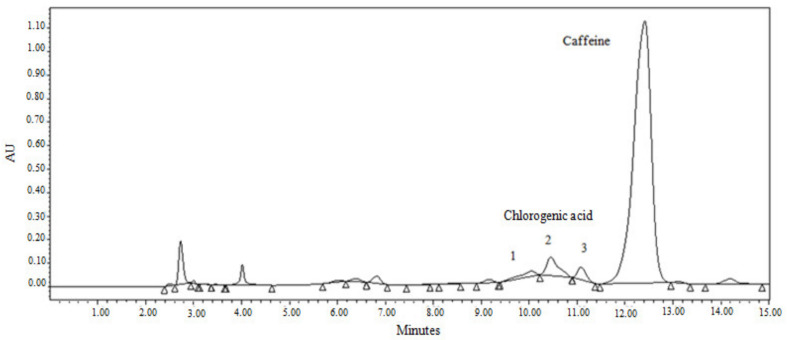
HPLC chromatogram of the supra extract for spent coffee grounds 1 (SCG1) subjected to the supramolecular method.

The chromatogram of the extract obtained (see Figure 1) revealed different chlorogenic acid isomers (peaks 1, 2 and 3) at retention times close to one of the standards (5-O-Caffeoylquinic acid, 5-CA). Spiking experiments with the chlorogenic acid standard (5-CA, 98%) were carried out to determine the peaks corresponding to the standard. The spiking consisted of replacing water with a certain volume of the chlorogenic acid standard solution. The comparison of the chromatograms with and without spiking showed that the chlorogenic acid standard has isomerized (due to its interaction with SCG) and increased the three peaks of the spiked sample instead of appearing as one single peak. The area of the first peak (peak 1) increased by 75%, while the second and third peaks (peaks 2 and 3) had a relative rise of roughly 29% and 31%, respectively. Therefore, all peaks were included in the measurement of CA content.

Neither the extraction of chlorogenic acid nor that of caffeine is selective (see Figure 1), which means that the extract obtained will have other components transferred from the coffee grounds.

**Figure 2:**
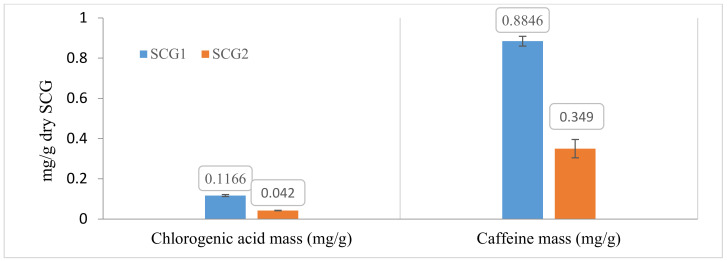
Chlorogenic acid and caffeine extracted with the supramolecular method (average of three replicates).

The results obtained are presented in Figure 2. Results are expressed as mg of bioactive substance (chlorogenic acid and caffeine) per g of dry SCG.

The experimental measurements have shown that SCG1 is richer in chlorogenic acid and caffeine than SCG2. Chlorogenic acid and caffeine obtained for SCG1 were 0.116 mg CA/g and 0.884 mg Caf/g, respectively.

Torres-Valenzuela et al. [30] have reported higher extraction values of chlorogenic acids and caffeine (4.3 mg CA/g and 3.32 mg Caf/g) using the same extraction method of SCG obtained from drip filter brewing of a Colombian 100% Arabica variety. The reasons for these differences may be mainly attributed to the type of coffee variety and coffee preparation method. As said before, coffee variety, genetic characteristics, agricultural processes, storage conditions, brewing time, and roasting degree can affect the content of CA and caffeine in SCG and their recovery rate [11,41,45,46].

In addition, a series of experiments were carried out to measure the affinity of the supra phase for chlorogenic acid compared to the inferior phase. The experiments were performed in triplicate without the use of coffee grounds but with the addition of approximately 1 mL of a 100 mg/L chlorogenic acid solution. The results demonstrate that only 34% of the total added chlorogenic acid was recovered in the supra phase, and 66% remained in the lower phase (inferior phase). This proved that the phase with the highest affinity for chlorogenic acid is the inferior phase.

Therefore, a quantification of the contents at the inferior phase (infra) was also measured to be able to compare the extraction content in both phases.

**Figure 3:**
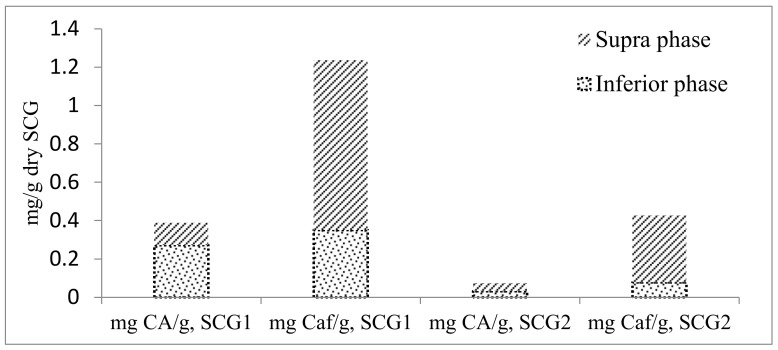
Chlorogenic acid and caffeine mass at the superior and inferior phases (average of three replicates) with the supramolecular solvent method.

As can be seen in Figure 3, a significant amount of chlorogenic acid is still present at the inferior phase, while for caffeine, the main quantity is found at the supra phase (although around 28% still remained at the inferior phase).

In terms of concentration, the supramolecular method extracted 48.7 mg/L of chlorogenic acid and 370 mg/L of caffeine at the superior phase from SCG1, while 277 mg/L of chlorogenic acid and 355 mg/L of caffeine concentrations were found at the inferior phase. Concentration results are important to know when direct use of the extract (without subsequent evaporation of the solvent) is possible for further industrial applications.

### 4.2. Extraction with Water Using Vortex

The influence of the solvent on the extraction results for chlorogenic acid and caffeine has been investigated using ultrapure water. Water is much cheaper and far more widely accessible than other solvents, such as dichloromethane and ethanol.

**Figure 4:**
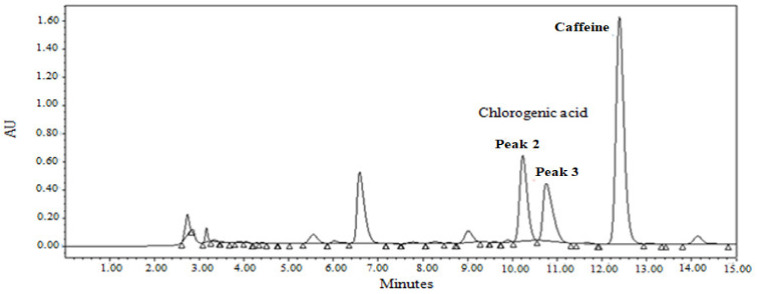
HPLC chromatogram of the water extract for spent coffee grounds 1 (SCG1) subjected to the water extraction method.

A comparison of spiked and non-spiked water extractions was also carried out. The objective of these experiments was to ensure which of the chlorogenic acid peaks corresponds to that of the standard. In the extractions with water, the first peak is practically negligible, while the others (peaks 2 and 3) present significant results in relation to the area (see Figure 4).

The effect of spiking promotes a simultaneous increase in both peaks of chlorogenic acid. The second peak of the spiked sample increased by a factor of 1.6, whereas the third peak increased by a factor of 1.2.

Therefore, to determine the amount of chlorogenic acid extracted in the coffee grounds samples, the sum of the two peak areas (peaks 2 and 3) has been used.

**Figure 5:**
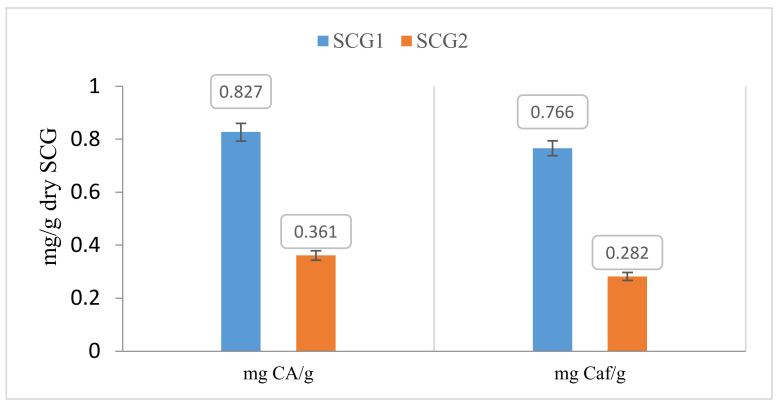
Extraction rate of chlorogenic acid mass and caffeine mass (average of three replicates) with the water extraction method.

Results also revealed that SCG1 is richer in chlorogenic acid and caffeine than SCG2 (see Figure 5). Chlorogenic acid and caffeine obtained for SCG1 were 0.827 mg CA/g and 0.766 mg Caf/g, respectively.

In terms of concentration in the extract obtained, water recovered 223 mg/L of chlorogenic acid and 207 mg/L of caffeine from SCG1.

### 4.3. Ultrasound-Assisted Water Extraction

Ultrasound-assisted water extraction at different temperatures (room temperature and 50 °C) was evaluated on chlorogenic acid and caffeine extraction.

**Figure 6:**
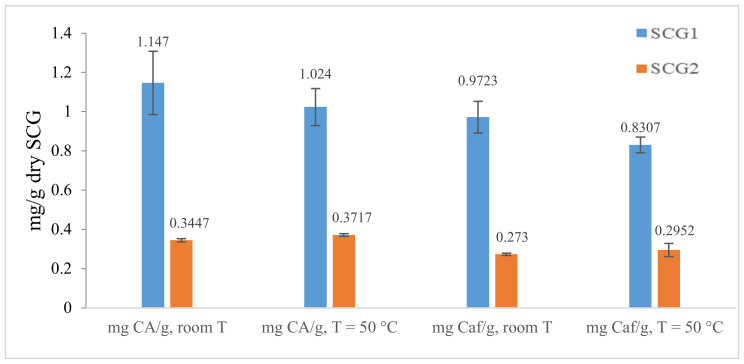
Extraction rate of chlorogenic acid mass (average of three replicates) using the ultrasound method at ambient temperature and at 50 °C.

The experiments did not show a variation in chlorogenic acid and caffeine extracted at 50 °C compared to room temperature for both spent coffee grounds. A slight positive effect was detected on SCG2 because the chlorogenic acid and caffeine masses were increased by about 11% and 12%, respectively, whereas for SCG1, a slight decrease was observed (about 11–14%). The best results were obtained at room temperature (1.15 mg CA/g and 0.972 mg Caf/g) (see Figure 6).

The effect of temperature on chlorogenic acid and caffeine is also discussed in the literature. Vandeponseele et al. [11] reported a small increase (+12%) of the caffeine mass (mg/g SCG) between 20 and 80 °C when using a mixture of H_2_O/EtOH (60/40 *v*/*v*) as extraction solvent, while Butiuk et al. [47] have found that a temperature between 30 and 80 °C did not affect the extraction content of chlorogenic acid from yerba mate stems when performed with water as the extraction solvent. However, when the temperature exceeds 80 °C, the amount of CA extracted decreases. Hence, we can infer that CA becomes unstable above a temperature of 80 °C.

In terms of concentration, ultrasound at ambient temperature extracted the most chlorogenic acid and caffeine from SCG1 with 332 mg/L and 282 mg/L, respectively, whereas ultrasound at 50 °C obtained only 284 mg/L and 231 mg/L, respectively.

### 4.4. Longer Extraction Time

The influence of the extraction time between 1 min and 5 min on the caffeine and chlorogenic acid results was investigated for two extraction methods: vortex-shaken with water and using supra solvents.

**Figure 7:**
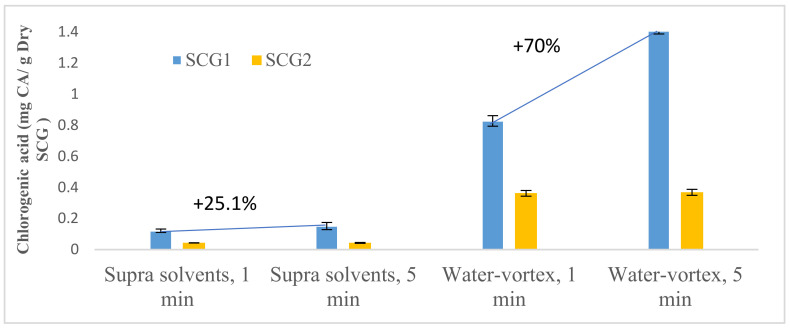
Influence of time for supra and water extraction of chlorogenic acid from spent coffee grounds.

**Figure 8:**
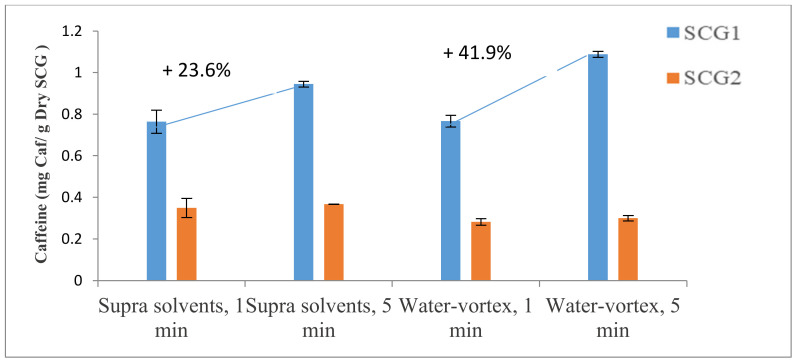
Influence of time for supra and water extraction of caffeine from spent coffee grounds.

The effect of the extraction time widely varies depending on the extraction method. For SCG1, an increase in chlorogenic acid mass of 25.1% and caffeine mass of 23.6% between 1 min and 5 min was associated with the extraction with supra, while there was an increase of 70% in the chlorogenic acid mass and 41.9% in the caffeine mass for the extraction with water, as can be seen in Figure 7 for the chlorogenic acid results and Figure 8 for caffeine.

For SCG2, the experiments revealed a very small increase in the chlorogenic acid (+0.3% for supra solvents or +1.8% for water) and the caffeine mass (+5% or +6%, respectively) between 1 min and 5 min.

According to Ballesteros-Gómez et al. [31], a few extraction minutes (5–10 min) are sufficient for liquid samples, while a longer extraction time is needed in the case of solid samples to accelerate and increase the mass transfer between the sample and the solvent. Thus, longer extraction times can probably be used in the present case if the aim is to extract as much chlorogenic acid and caffeine as possible.

### 4.5. Comparison of the Different Extraction Methods Tested

**Figure 9:**
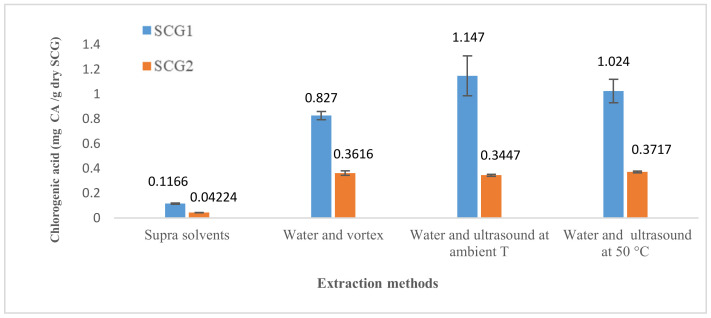
Chlorogenic acid mass extracted as a function of the different extraction methods.

**Figure 10:**
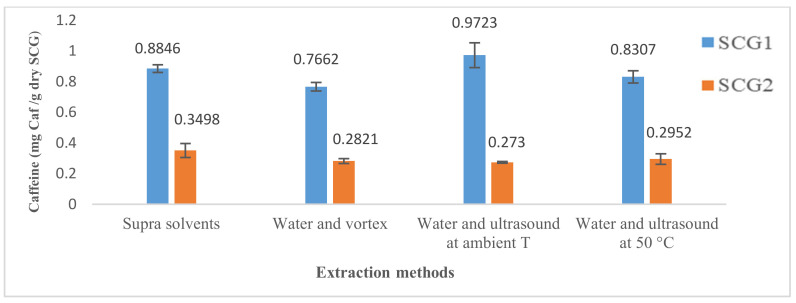
Caffeine mass extracted as a function of the different extraction methods.

The results comparing the different methods to extract chlorogenic acid and caffeine are presented in Figure 9 and Figure 10, respectively.

A comparison of the results indicates significant differences between the bioactive substances extracted (chlorogenic acid and caffeine) and the type of coffee. On the other hand, remarkable differences were also detected among all the extraction methods examined (Figure 9 and Figure 10).

SCG1 showed the highest extraction values, which may be attributed to the characteristics of the coffee variety, the proportions in the coffee blend used, the manufacturing process (roasting) and the coffee preparation method. As said before, Robusta has about twice the caffeine content as Arabica and is slightly richer in chlorogenic acid [19]. In the present case, SCG1 was less roasted than SCG2, and both are mixtures with unknown proportions of Arabica and Robusta.

The comparison of methods that are discussed below refers just to SCG1, the one showing better results.

The method that led to the best results was the extraction with ultrasound at room temperature, while extraction of supra solvents was, surprisingly, not the best. Nevertheless, ultrasounds at room temperature present a higher coefficient of variation, which means that the results of the triplicate have more dispersion (14% and 8.3% for chlorogenic acid and caffeine, respectively).

For chlorogenic acid, water extraction assisted by ultrasound at room temperature yielded the greatest amount with 1.15 mg CA/g, followed by ultrasound at 50 °C and then extraction with water using a vortex. This best result was seven times higher than the one obtained by supramolecular solvent extraction.

For caffeine, ultrasound at room temperature also demonstrated the best results with 0.972 mg Caf/g, followed by supramolecular solvent extraction (0.885 mg Caf/g).

Supramolecular solvent extraction did not succeed at extracting higher contents of bioactive components, especially chlorogenic acid, in comparison with the conventional methods. This can be attributed either to the distribution of analytes between supra and infra phases or the thermodynamic difficulty in obtaining “supra” solvents (which require self-assembly and coacervation processes that are spontaneous and sequential).

## 5. Environmental Comparison: Methodology and Results

The extraction methods to be compared will be the supramolecular solvent method (of greater novelty) and the water (vortex-shaken) method (of easier industrial scaling). The extraction yields obtained for chlorogenic acid (CA) were 0.827 mg CA/g SCG and 0.116 mg CA/g SCG using water and supramolecular extraction methods, respectively, while for caffeine (Caf), yields were 0.766 mg Caf/g SCG and 0.885 mg Caf/g SCG, respectively (see Section 4.5).

In order to perform an environmental comparison using the life cycle assessment (LCA) methodology, we need to compare products/processes performing identical functions. In the present study, the extraction methods were compared for the manufacture of the same commercial product. Two different commercial products have been chosen for comparison: one of them is a cosmetic face cream containing chlorogenic acid (CA) because the antioxidant property (from polyphenols) is the one required in the product, and the other is an eye contour serum containing caffeine due to its anti-inflammatory property.

The LCA methodology, as specified by ISO 14,044 [48], was used for the environmental comparison. The systems were modeled using the software Gabi v10.6.135, and the impact categories used for the environmental assessment were the ones suggested by the EF 3.0 method. Thirteen impact categories were evaluated, and all of them were classified into three types: damage to health, damage to ecosystems, and resource consumption.

### 5.1. Environmental Comparison for the Manufacture of a Face Cosmetic Cream

#### 5.1.1. Chlorogenic Acid Recovery: Scope and Inventory Data

As said before, in cosmetic cream, the antioxidant property of chlorogenic acid (and other polyphenols) is the property required.

Thus, the Functional Unit (FU) chosen for this comparison was “125 mg of chlorogenic acid needed for 100 g of a face cosmetic cream”.

The amount of chlorogenic acid in the cream was obtained by the relation between CA and the total phenolic compounds, as the cream manufacturing laboratory reported a 4% content of total phenolic compounds in the cream. Thus, the proportion of chlorogenic acid related to the total polyphenols in an SCG extract was estimated by Xu et al. [29], where the authors reported that chlorogenic acid represents 3.12% of the total phenolic compounds found in a specific SCG. A content of 125 mg of chlorogenic acid per 100 g of cream was calculated using this percentage as the FU of the present study.

**Figure 11:**
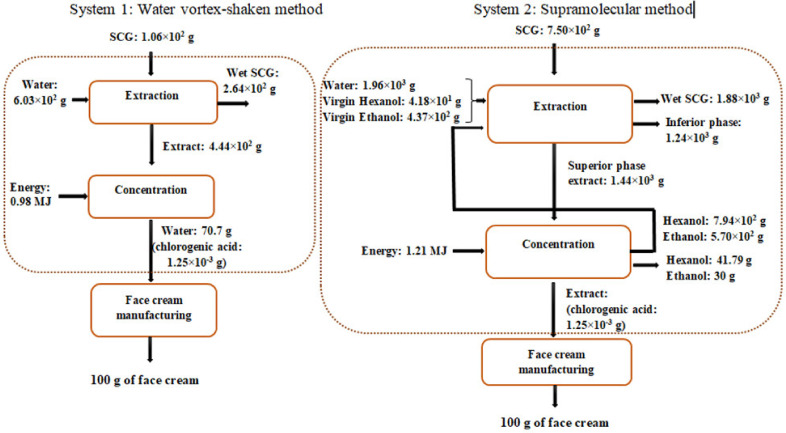
System boundaries of the LCA study to obtain a face cream.

System boundaries of the LCA comparison, as well as the inventory data, are illustrated in Figure 11. The system boundaries of the study are from the extraction process to the concentration of the extract just before its incorporation into the face cream. The extraction process consists of the extraction of CA (and caffeine) from SCG, followed by filtration to separate the extract from the solid. The concentration process involved the complete evaporation of ethanol and 1-hexanol in System 2 or the partial evaporation of water (taking into account the percentage of water, 70.7%, required in the composition of the cream) in System 1. The evaporated 1-hexanol and ethanol were reused as inputs for the extraction process. A reusing efficiency of 95% was estimated for this study, and the other 5% of the spent solvents were treated in an incineration plant.

The stages studied were those that varied in the two systems and excluded the ones that intervened in the same way (such as the energy used in the vortex shaker, centrifuge, and filtration).

After extraction, the remaining wet SCG and the inferior phase (in System 2) were treated in a composting plant and a wastewater treatment facility, respectively.

The inventory data was obtained through experiments on a laboratory scale where a 0.7 g sample of dry spent coffee grounds was mixed with 4 mL of solvent. The solvent is water for System 1 and a mixture of 1.2 mL ethanol, 0.96 mL 1-hexanol, and 1.84 mL water for System 2.

After the extraction and for both methods, 60% humidity for SCG was considered (so the amount of wet SCG is 1.75 g from 0.7 g of dried SCG). The mass of the extract for System 1 consisted of the remaining water calculated according to the mass balance, while for System 2, the mass of the extract was calculated based on the volume of the supra phase obtained (1.67 mL from 0.7 g dry SCG), assuming this phase contains all of the 1-hexanol (0.96 mL) and part of the ethanol. The volume of ethanol is the total volume of the extract minus the volume of 1-hexanol. Subsequently, the mass of the different solvents (water and ethanol) in the inferior phase (going to wastewater treatment) was determined by applying the mass balance.

According to the experiments carried out in the laboratory, 0.827 mg CA/g SCG and 0.116 mg CA/g SCG were obtained using water and supramolecular extraction methods, respectively. These results were used to determine the different inputs and outputs to obtain 125 mg CA/FU.

The thermal energy required for each system was theoretically calculated by taking into account the energy required to heat the solvent until boiling point plus the energy required to evaporate this solvent.

#### 5.1.2. Chlorogenic Acid Recovery: Impact Assessment and Discussion of the Results

**Figure 12:**
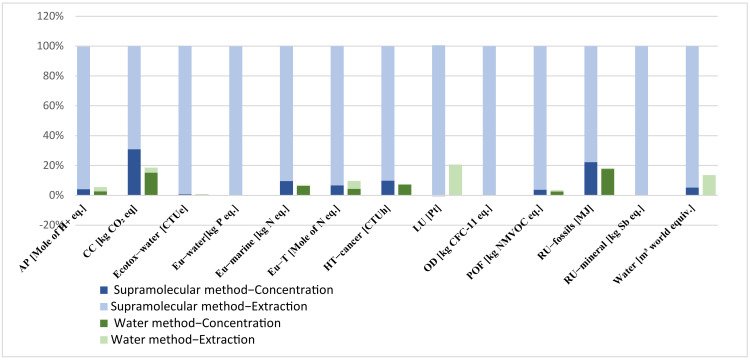
Environmental impacts of chlorogenic acid extraction using water and supra methods. AP: acidification; CC: climate change; Ecotox−water: freshwater ecotoxicity; Eu−water: freshwater eutrophication; Eu−marine: marine eutrophication; Eu−T: terrestrial eutrophication; HT−cancer: human toxicity, cancer; LU: land use; OD: ozone depletion; POF: photochemical ozone formation; RU−fossils: resource use, fossils; RU−mineral: resource use, mineral and metals; and Water: water use.

Results in Figure 12 reveal that water extraction is the eco-friendliest method to obtain 125 mg of CA (the amount needed to produce 100 g of face cosmetic cream), with lower impacts compared to the supramolecular extraction method. Impacts on System 1 are especially linked to the use of thermal energy in the concentration stage (except for land use, LU, and water consumption, Water), while, for the supramolecular method, the impacts are mainly attributed to the lower extraction yield of CA and to the production of the solvents consumed in the extraction stage, since 1-hexanol and ethanol have higher impacts than water.

### 5.2. Environmental Comparison for the Production of an Eye Contour Serum

#### 5.2.1. Caffeine Recovery: Scope and Inventory Data

In the case of an eye contour serum, the anti-inflammatory property of the caffeine is appreciated. Thus, the Functional Unit (FU) chosen for the comparison was “5 g of caffeine needed for 100 g of an eye contour serum”, as the serum contains 5% of caffeine (content specified in commercial products).

**Figure 13:**
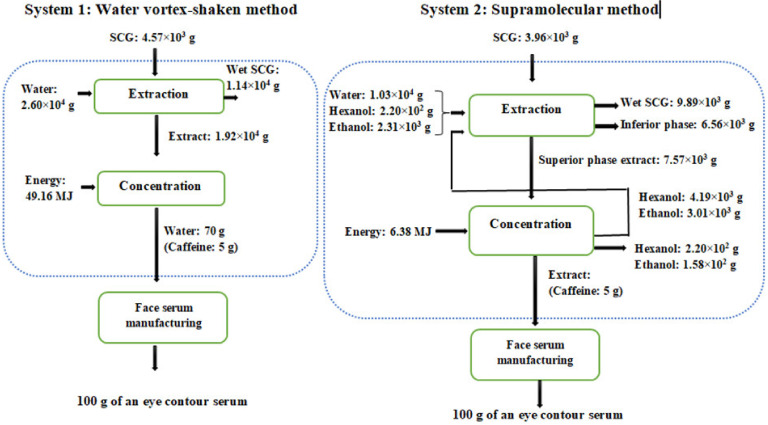
System boundaries of the LCA study to obtain an eye contour serum.

System boundaries of the LCA comparison, as well as the inventory data, are shown in Figure 13. The system boundaries of the study are the same as before, from the extraction process to the concentration of the extract, just before its incorporation into the serum. The extraction process consists of the extraction of caffeine from SCG, followed by separation to isolate the extract from the solid. The concentration process involves the complete evaporation of ethanol and 1-hexanol in System 2 and partial evaporation of water (taking into account the 70% content of water in the serum).

The same hypothesis as before was taken, where 95% of 1-hexanol and ethanol is reused and 5% not reused is incinerated, wet SCG contains 60% humidity and is composted, and finally, wastewater is treated in a wastewater treatment plant.

Inventory data were obtained in the same way explained in Section 5.1.1 using the yield of caffeine recovery obtained in the laboratory (see Section 4.5): a total of 0.766 mg Caf/g SCG and 0.885 mg Caf/g SCG for water and supramolecular extraction methods, respectively.

#### 5.2.2. Caffeine Recovery: Impact Assessment and Discussion of the Results

**Figure 14:**
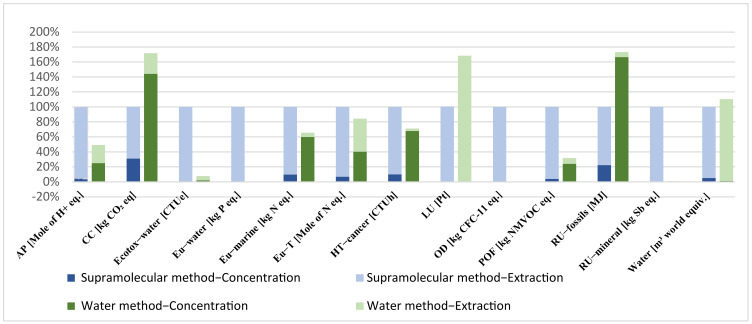
Environmental impacts of caffeine extraction using water and supra methods. AP: acidification; CC: climate change; Ecotox−water: freshwater ecotoxicity; Eu−water: freshwater eutrophication; Eu−marine: marine eutrophication; Eu−T: terrestrial eutrophication; HT−cancer: human toxicity, cancer; LU: land use; OD: ozone depletion; POF: photochemical ozone formation; RU−fossils: resource use, fossils; RU−mineral: resource use, mineral and metals; and Water: water use.

Results in Figure 14 revealed that water extraction is still environmentally better to obtain 5 g of caffeine (the amount needed to produce 100 g of eye serum), with generally lower impacts compared with the supramolecular extraction method (except for climate change, CC; land use, LU; fossil resources use, RU−fossils; and water use, Water).

## 6. Conclusions and Recommendations

A review of the literature on chlorogenic acid and caffeine extraction methods from spent coffee grounds (SCGs) was conducted. Results obtained by different studies are difficult to compare due to the differences in coffee variety, roasting degree, and coffee preparation method. Most authors have optimized a specific extraction method in terms of time, temperature, and solvent/solid ratio. Authors usually do not compare different extraction methods and solvents using the same SCG. In order to generate comparative information, in the present paper, four different extraction methods were compared using the shortest extraction time and lowest solvent/solid ratio (1 min and 5.7 mL/g) described in the literature. Supramolecular solvent extraction (vortex-shaken) and water (vortex-shaken) using ultrasound at two different temperatures were experimentally tested.

Two different SCGs were extracted: SCG1 (“Novell Gourmet Responsable”) and SCG2 (“MamaSame”). Results revealed significant differences between the two brands, which are attributed to their origin, roasting degree, and coffee infusion preparation, with SCG1 (which is a lighter roast) being richer in chlorogenic acid and caffeine. Therefore, results are discussed for SCG1.

Ultrasound at room temperature using water as a solvent was demonstrated to be the most effective method to recover chlorogenic acid and caffeine with 1.15 mg CA/g and 0.972 mg Caf/g, respectively. While the supramolecular solvent is more selective for caffeine, obtaining a concentration of 370 mg Caf/L in the extract compared to 49 mg/L for CA.

Additionally, the influence of the extraction time, between 1 min and 5 min, on the extraction yield of chlorogenic acid and caffeine revealed that a longer extraction time (5 min) helps to extract higher content of chlorogenic acid and caffeine (a +70% and +42% increase, respectively).

When the supramolecular solvent method is environmentally compared with water extraction (vortex-shaken) using LCA methodology, the results show that water extraction is usually better for both chlorogenic acid and caffeine extraction. This is mainly due to the impact of the production of solvents (ethanol and 1-hexanol) used in the supra solvent method and to the lower amount of the analyte extracted (especially in the case of chlorogenic acid). It is worth mentioning that lengthening the extraction time (from 1 to 5 min) increases the water extraction yield much more than the supramolecular yield, and, in addition, water extraction would be easier to implement at an industrial scale. The results show that when the water extraction yield is similar to the yield obtained with other solvents, water will usually be the preferred solvent from an environmental perspective. The supramolecular method will certainly be efficient to use and environmentally preferable when extracting other types of analytes having less affinity for water.

The results presented here are intended to promote the circularity of SCG by obtaining valuable substances, such as caffeine and chlorogenic acid. There is no standard method to extract those analytes from SCG; therefore, a large number of alternatives are presented in the literature. Surprisingly, water, which delivers better results than those new alternatives, is not discussed in the literature, which, until now, has not presented comparative studies of different extraction methods. Our proposal is that when a new extraction alternative is reported, comparative studies are needed to evaluate both extraction yields and environmental performance in comparison with older methods.

## Data Availability

Data is contained within the article. Nevertheless, if more details are needed they will be available from the corresponding author.

## References

[1] Lang L., Wang Y., Chen X., Zhang Z., Yang N., Xue B., Han W. (2020). Awareness of Food Waste Recycling in Restaurants: Evidence from China. Resour. Conserv. Recycl..

[2] Ishangulyyev R., Kim S., Lee S.H. (2019). Understanding Food Loss and Waste—Why Are We Losing and Wasting Food?. Foods.

[3] European Commission Communication from the Commission to the European Parliament, the Council, the European Economic and Social Committee and the Committee of the Regions. https://eur-lex.europa.eu/legal-content/EN/TXT/?qid=1551965345008&uri=CELEX:32018L0852.

[4] Food and Agriculture Organization of the United Nations (2021). Food Waste Index Report 2021.

[5] Wu T.-W., Zhang H., Peng W., Lü F., He P.-J. (2023). Applications of Convolutional Neural Networks for Intelligent Waste Identification and Recycling: A Review. Resour. Conserv. Recycl..

[6] Ferdous W., Manalo A., Siddique R., Mendis P., Zhuge Y., Wong H.S., Lokuge W., Aravinthan T., Schubel P. (2021). Recycling of Landfill Wastes (Tyres, Plastics and Glass) in Construction—A Review on Global Waste Generation, Performance, Application and Future Opportunities. Resour. Conserv. Recycl..

[7] De Sadeleer I., Brattebø H., Callewaert P. (2020). Waste Prevention, Energy Recovery or Recycling—Directions for Household Food Waste Management in Light of Circular Economy Policy. Resour. Conserv. Recycl..

[8] Conde T., Mussatto S.I. (2016). Isolation of Polyphenols from Spent Coffee Grounds and Silverskin by Mild Hydrothermal Pretreatment. Prep. Biochem. Biotechnol..

[9] International Coffee Organization. https://www.ico.org/.

[10] Gouws S., Muller M. (2021). Valorization of Products from Grounded-Coffee Beans. Sci. Rep..

[11] Vandeponseele A., Draye M., Piot C., Chatel G. (2021). Study of Influential Parameters of the Caffeine Extraction from Spent Coffee Grounds: From Brewing Coffee Method to the Waste Treatment Conditions. Clean Technol..

[12] Shang Y.F., Xu J.L., Lee W.J., Um B.H. (2017). Antioxidative Polyphenolics Obtained from Spent Coffee Grounds by Pressurized Liquid Extraction. South African J. Bot..

[13] Silva M.d.O., Honfoga J.N.B., Medeiros L.L.d., Madruga M.S., Bezerra T.K.A. (2020). Obtaining Bioactive Compounds from the Coffee Husk (*Coffea Arabica* L.) Using Different Extraction Methods. Molecules.

[14] Perta-Crisan S., Ursachi C., Munteanu F.-D. (2019). Trends in Valorisation of Spent Coffee Grounds: A Review. Sci. Tech. Bull. Ser. Chem. Food Sci. Eng..

[15] Andrade K.S., Gonalvez R.T., Maraschin M., Ribeiro-Do-Valle R.M., Martínez J., Ferreira S.R.S. (2012). Supercritical Fluid Extraction from Spent Coffee Grounds and Coffee Husks: Antioxidant Activity and Effect of Operational Variables on Extract Composition. Talanta.

[16] Gemechu F.G. (2020). Embracing Nutritional Qualities, Biological Activities and Technological Properties of Coffee Byproducts in Functional Food Formulation. Trends Food Sci. Technol..

[17] Cruz Velasquez S.M. (2018). Evaluación de La Actividad Antioxidante y Detección de Marcadores Químicos En Extractos de Hojas y Granos de Siete Variedades de Café Comercializadas En Guatemala.

[18] Zuorro A., Lavecchia R. (2012). Spent Coffee Grounds as a Valuable Source of Phenolic Compounds and Bioenergy. J. Clean. Prod..

[19] Panusa A., Zuorro A., Lavecchia R., Marrosu G., Petrucci R. (2013). Recovery of Natural Antioxidants from Spent Coffee Grounds. J. Agric. Food Chem..

[20] Mussatto S.I., Ballesteros L.F., Martins S., Teixeira J.A. (2011). Extraction of Antioxidant Phenolic Compounds from Spent Coffee Grounds. Sep. Purif. Technol..

[21] Andrade K.S. (2012). Avaliação Das Técnicas de Extração e Do Potencial Antioxidante Dos Extratos Obtidos a Partir de Casca e de Borra de Café (*Coffea Arabica* L.). Master’s Thesis.

[22] Acevedo F., Rubilar M., Scheuermann E., Cancino B., Uquiche E., Garcés M., Inostroza K., Shene C. (2013). Spent Coffee Grounds as a Renewable Source of Bioactive Compounds. J. Biobased Mater. Bioenergy.

[23] Ballesteros L.F., Ramirez M.J., Orrego C.E., Teixeira J.A., Mussatto S.I. (2017). Optimization of Autohydrolysis Conditions to Extract Antioxidant Phenolic Compounds from Spent Coffee Grounds. J. Food Eng..

[24] Caballero-Galván A.S., Restrepo-Serna D.L., Ortiz-Sánchez M., Cardona-Alzate C.A. (2018). Analysis of Extraction Kinetics of Bioactive Compounds from Spent Coffee Grounds (Coffea Arábica). Waste Biomass Valorization.

[25] Al-Dhabi N.A., Ponmurugan K., Maran Jeganathan P. (2017). Development and Validation of Ultrasound-Assisted Solid-Liquid Extraction of Phenolic Compounds from Waste Spent Coffee Grounds. Ultrason. Sonochem..

[26] Arauzo P.J., Lucian M., Du L., Olszewski M.P., Fiori L., Kruse A. (2020). Improving the Recovery of Phenolic Compounds from Spent Coffee Grounds by Using Hydrothermal Delignification Coupled with Ultrasound Assisted Extraction. Biomass Bioenergy.

[27] Pavlović M.D., Buntić A.V., Šiler-Marinković S.S., Dimitrijević-Branković S.I. (2013). Ethanol Influenced Fast Microwave-Assisted Extraction for Natural Antioxidants Obtaining from Spent Filter Coffee. Sep. Purif. Technol..

[28] Pettinato M., Casazza A.A., Ferrari P.F., Palombo D., Perego P. (2019). Eco-Sustainable Recovery of Antioxidants from Spent Coffee Grounds by Microwave-Assisted Extraction: Process Optimization, Kinetic Modeling and Biological Validation. Food Bioprod. Process..

[29] Xu H., Wang W., Liu X., Yuan F., Gao Y. (2015). Antioxidative Phenolics Obtained from Spent Coffee Grounds (Coffea Arabica L.) by Subcritical Water Extraction. Ind. Crops Prod..

[30] Torres-Valenzuela L.S., Ballesteros-Gómez A., Sanin A., Rubio S. (2019). Valorization of Spent Coffee Grounds by vecular Solvent Extraction. Sep. Purif. Technol..

[31] Ballesteros-Gómez A., Sicilia M.D., Rubio S. (2010). Supramolecular Solvents in the Extraction of Organic Compounds. A Review. Anal. Chim. Acta.

[32] Clifford M.N., Wight J. (1976). The Measurement of Feruloylquinic Acids and Caffeoylquinic Acids in Coffee Beans. Development of the Technique and Its Preliminary Application to Green Coffee Beans. J. Sci. Food Agric..

[33] Singleton V.L., Rossi J.A. (1965). Colorimetry of Total Phenolics with Phosphomolybdic-Phosphotungstic Acid Reagents. Am. J. Enol. Vitic..

[34] Kyoung Chun O., Kim D.O. (2004). Consideration on Equivalent Chemicals in Total Phenolic Assay of Chlorogenic Acid-Rich Plums. Food Res. Int..

[35] Malta L.G., Liu R.H. (2014). Analyses of Total Phenolics, Total Flavonoids, and Total Antioxidant Activities in Foods and Dietary Supplements. Encyclopedia of Agriculture and Food Systems.

[36] Campos-Vega R., Loarca-Piña G., Vergara-Castañeda H.A., Dave Oomah B. (2015). Spent Coffee Grounds: A Review on Current Research and Future Prospects. Trends Food Sci. Technol..

[37] Ramón-Gonçalves M., Gómez-Mejía E., Rosales-Conrado N., León-González M.E., Madrid Y. (2019). Extraction, Identification and Quantification of Polyphenols from Spent Coffee Grounds by Chromatographic Methods and Chemometric Analyses. Waste Manag..

[38] Shalmashi A., Golmohammad F. (2010). Solubility of Caffeine in Water, Ethyl Acetate, Ethanol, Carbon Tetrachloride, Methanol, Chloroform, Dichloromethane, and Acetone between 298 and 323 K. Lat. Am. Appl. Res..

[39] Okur I., Soyler B., Sezer P., Oztop M.H., Alpas H. (2021). Improving the Recovery of Phenolic Compounds from Spent Coffee Grounds (SCG) by Environmentally Friendly Extraction Techniques. Molecules.

[40] Iziar A.L., Mena P., Calani L., Cid C., Del Rio D., Lean M.E., Crozier A. (2014). Variations in Caffeine and Chlorogenic Acid Contents of Coffees: What Are We Drinking?. Food Funct..

[41] Mills C.E., Oruna-Concha M.J., Mottram D.S., Gibson G.R., Spencer J.P.E. (2013). The Effect of Processing on Chlorogenic Acid Content of Commercially Available Coffee. Food Chem..

[42] Vignoli J.A., Bassoli D.G., Benassi M.T. (2011). Antioxidant Activity, Polyphenols, Caffeine and Melanoidins in Soluble Coffee: The Influence of Processing Conditions and Raw Material. Food Chem..

[43] Jeon J.S., Kim H.T., Jeong I.H., Hong S.R., Oh M.S., Yoon M.H., Shim J.H., Jeong J.H., Abd El-Aty A.M. (2019). Contents of Chlorogenic Acids and Caffeine in Various Coffee-Related Products. J. Adv. Res..

[44] Kulapichitr F., Borompichaichartkul C., Fang M., Suppavorasatit I., Cadwallader K.R. (2022). Effect of Post-Harvest Drying Process on Chlorogenic Acids, Antioxidant Activities and CIE-Lab Color of Thai Arabica Green Coffee Beans. Food Chem..

[45] McCusker R.R., Goldberger B.A., Cone E.J. (2003). Caffeine Content of Specialty Coffees. J. Anal. Toxicol..

[46] Caprioli G., Cortese M., Sagratini G., Vittori S. (2015). The Influence of Different Types of Preparation (Espresso and Brew) on Coffee Aroma and Main Bioactive Constituents. Food Sci. Nutr..

[47] Butiuk A.P., Maidana S.A., Adachi O., Akakabe Y., Martos M.A., Hours R.A. (2021). Optimization and Modeling of the Chlorogenic Acid Extraction from a Residue of Yerba Mate Processing. J. Appl. Res. Med. Aromat. Plants.

[48] (2006). Environmental Management: Life Cycle Assessment; Principles and Framework.

